# Systems Thinking as a Framework for Analyzing Commercial Determinants of Health

**DOI:** 10.1111/1468-0009.12339

**Published:** 2018-09-11

**Authors:** CÉCILE KNAI, MARK PETTICREW, NICHOLAS MAYS, SIMON CAPEWELL, REBECCA CASSIDY, STEVEN CUMMINS, ELIZABETH EASTMURE, PATRICK FAFARD, BENJAMIN HAWKINS, JØRGEN DEJGÅRD JENSEN, SRINIVASA VITTAL KATIKIREDDI, MODI MWATSAMA, JIM ORFORD, HEIDE WEISHAAR

**Affiliations:** ^1^ London School of Hygiene and Tropical Medicine; ^2^ University of Liverpool; ^3^ Goldsmiths, University of London; ^4^ University of Ottawa; ^5^ University of Copenhagen; ^6^ University of Glasgow; ^7^ UK Health Forum; ^8^ University of Birmingham; ^9^ Hertie School of Governance

**Keywords:** systems thinking, noncommunicable diseases, unhealthy commodity industries

## Abstract

**Policy Points:**

- Worldwide, more than 70% of all deaths are attributable to noncommunicable diseases (NCDs), nearly half of which are premature and apply to individuals of working age. Although such deaths are largely preventable, effective solutions continue to elude the public health community.
- One reason is the considerable influence of the “commercial determinants of health”: NCDs are the product of a system that includes powerful corporate actors, who are often involved in public health policymaking.
- This article shows how a complex systems perspective may be used to analyze the commercial determinants of NCDs, and it explains how this can help with (1) conceptualizing the problem of NCDs and (2) developing effective policy interventions.

**Context:**

The high burden of noncommunicable diseases (NCDs) is politically salient and eminently preventable. However, effective solutions largely continue to elude the public health community. Two pressing issues heighten this challenge: the first is the public health community's narrow approach to addressing NCDs, and the second is the involvement of corporate actors in policymaking. While NCDs are often conceptualized in terms of individual‐level risk factors, we argue that they should be reframed as products of a complex system. This article explores the value of a systems approach to understanding NCDs as an emergent property of a complex system, with a focus on commercial actors.

**Methods:**

Drawing on Donella Meadows's systems thinking framework, this article examines how a systems perspective may be used to analyze the commercial determinants of NCDs and, specifically, how unhealthy commodity industries influence public health policy.

**Findings:**

Unhealthy commodity industries actively design and shape the NCD policy system, intervene at different levels of the system to gain agency over policy and politics, and legitimize their presence in public health policy decisions.

**Conclusions:**

It should be possible to apply the principles of systems thinking to other complex public health issues, not just NCDs. Such an approach should be tested and refined for other complex public health challenges.

Reversing the high burden of noncommunicable diseases (NCDs) such as cardiovascular diseases, cancers, diabetes, and mental illness is a key global health challenge. The latest estimates by the World Health Organization (WHO) indicate that approximately 70% of all deaths worldwide are attributable to NCDs, nearly half of which are premature and in individuals of working age.^1^

Despite significant global commitments^2^ and the fact that NCDs are eminently preventable,^3^, ^4^ effective solutions to reducing NCDs^5^, ^6^, ^7^, ^8^ largely continue to elude the public health community. Two issues make responding to this challenge particularly difficult. The first is the public health community's mainly narrow approach to solving public health problems (such as NCDs). The second issue is the involvement of corporate actors in public health policymaking. This article addresses the first issue by exploring the value of a systems approach to understanding NCDs as an emergent property of a complex system. It addresses the second issue by focusing on the influence of commercial actors within that system.

## Narrow Public Health Approaches to Solving Public Health Problems

The etiology of NCDs is complex and multifactorial, influenced by a range of individual, social, environmental, political, cultural, economic, and commercial determinants. Although this is widely acknowledged in the literature,^9^ as is the need for more “upstream” solutions (such as alcohol pricing policies^10^ and policies to address the nutrition transition and corresponding double burden of disease),^11^ the predominant response continues to be isolated, “downstream” interventions. These tend to focus on education and individual‐level behavior change despite the evidence that most of these approaches are effective only in the short term.^12^ The public health community continues to rely on downstream interventions because evidence of their effectiveness is more readily available than that for upstream interventions, a situation known as the “inverse evidence law.”^13^ Interventions at the population level (eg, national policies or large‐scale area‐based changes) are more likely to affect multiple health and nonhealth outcomes.^14^ However, their scale and other ethical and methodological challenges in their evaluation almost always militate against the conduct of so‐called gold‐standard approaches to evidence generation such as randomized controlled trials (RCTs).^15^ Thus other methodological approaches, such as evaluations of natural experiments, are more often used.^16^ Common to both designs is the idea of a counterfactual: attempting to isolate the causal effect of a single intervention on a single outcome of interest, holding all other factors constant. The complex etiology of NCDs, however, creates an epistemological challenge for evaluating programs designed to reduce them. Given that NCDs are the result of a complex set of factors^3^ operating at several levels, from the individual to the societal, there are 2 key problems for which traditional evaluation approaches do not adequately account: complex causality and multiple outcomes.

### Complex Causality

Commercial influences, international trade agreements, agricultural and food policies, and other macrolevel factors may have both direct and indirect influences on NCDs as well as contributory factors such as smoking, inactivity, and obesity. These influences operate through long and complex causal pathways. They can also interact with one another and their contexts, with implications for interventions at different levels. For these reasons, direct causal pathways between, for example, the marketing of sugar‐sweetened beverages to children and child obesity outcomes are difficult to identify. Similarly, causality in relation to the effectiveness of specific interventions (eg, restrictions on advertising to children) is more difficult to show than for individual interventions that are amenable to RCTs.

### Multiple Outcomes

While it is plausible to focus on, for example, body mass index (BMI) or weight as one primary outcome measure when trying to reduce the burden of NCDs, changes in health outcomes are not the intended, and typically are not the sole, goal of most policies that have the potential to influence the upstream determinants of NCDs. For example, the agricultural policy of the European Union (EU) has a wide range of goals, such as producing food, protecting rural communities and the environment, protecting farmers’ incomes, and promoting employment. Nevertheless, the EU's agricultural policy is also likely to have an important if distant effect on health outcomes. Evaluating the effectiveness of interventions based on a single or even a few primary health outcomes may therefore be misleading, particularly because the effects on public health outcomes may be mediated through diverse nonhealth pathways. Conducting evaluations from a systems perspective, therefore, offers an opportunity to address this evidence gap, as such evaluations can incorporate the assessment of change in a wider range of health and nonhealth outcomes. We next describe both what this might involve in practice and the potential it offers for understanding the commercial determinants of health.

## The Involvement of Corporate Actors in Public Health Policymaking

The second challenge in responding positively to NCDs (and the focus of this article) lies in the involvement of corporate actors in public health policymaking. This has been characterized by a shift in focus to multiple (sometimes overlapping) decision‐making forums within a complex system of multilevel governance. The range of actors participating in decision making has diversified,^17^ echoing broader shifts in the nature of government.^18^, ^19^ The adverse influence of corporate actors in public health policy—specifically in areas such as alcohol, tobacco, food and nutrition, and gambling—is well documented and there is a coherence of approaches across these industries.^18^, ^20^, ^21^, ^22^ Unhealthy commodity industries (UCIs) are defined as industries in which a significant share of their product portfolio comprises unhealthy products including tobacco, alcohol, energy‐dense and low‐nutrient foods and beverages, and gambling services. Although the gambling industry has received less attention than other UCIs as a driver of NCDs, it is expanding rapidly and is associated with other risk factors for NCDs (eg, alcohol consumption).^23^, ^24^ Disabilities associated with gambling harms are now comparable to those associated with alcohol misuse, with even low‐level gambling being potentially harmful.^25^

The strategies and approaches that UCIs use to promote their products and choices that are detrimental to health^26^ include strategies often categorized as “market” and “nonmarket” components,^27^ although the distinction between them is increasingly debatable. In addressing the dual forces of increasing globalization and corporations’ tailoring of global strategies to local conditions, transnational corporations have become expert at “glocalization,” or the creation of demand by applying a tailored “marketing mix” (including price, placement, promotions, and product), leveraging their global brands, implementing different activities in different market types, and segmenting the target market.^28^, ^29^ Nonmarket strategies, whose purpose is to create additional value for businesses by improving overall perceptions of the business,^20^, ^21^, ^30^ aim to leverage political and social influence, so‐called soft power, to influence policy.^21^ These strategies include influencing the creation of evidence; questioning the effectiveness of statutory regulation and emphasizing self‐regulation and public‐private partnerships; publicly discrediting researchers; focusing on individual responsibility; attempting to frame the extent and nature of alcohol‐related harms and the relevant solutions; and forming alliances with other sectors or the public to give the impression of larger support for the industry's position.^30^, ^31^ Miller and Harkins further explain the scope of corporate political influence by warning that “it is no longer enough to think about corporations only as attempting to influence policy. In reality, much decision‐making power has been directly devolved to them while corporations are increasingly ‘internal’ to the State.”^18^ This is a fundamental point: public health actors typically continue to treat corporations as external to the state because they have a fiduciary responsibility to maximize shareholder value through the pursuit of profit, an end goal different from those of public policy. However, this fails to recognize “the corporatization of public health,”^32^ the fact that corporations are increasingly part of the fabric of public health policymaking, as just explained. This is compounded by the fact that many government activities have now devolved to arm's‐length organizations, including those delivered by corporations, with the potential for attendant conflicts of interest.^33^, ^34^, ^35^

The involvement of UCIs in public policy, in particular via public‐private partnerships and self‐regulation mechanisms, could be an effective way of improving NCDs, although a growing body of literature consistently demonstrates that they are not or that they are unlikely to do so; they are usually supported by a weak evidence base while protecting business interests.^10^, ^36^, ^37^, ^38^, ^39^, ^40^, ^41^, ^42^, ^43^, ^44^, ^45^, ^46^, ^47^ Against this, UCIs reject the most effective interventions to improve public health (including changes in pricing, promotion, or supply that would affect sales and profits). Moreover, self‐regulatory regimes and corporate social responsibility (CSR) programs help build constituencies across sectors and beyond core business activities and are thus very effective strategies to create consumers’ brand associations, increase brand recall, and shape purchase intentions.^48^ Finally, strategies that influence the creation or presentation of evidence have been shown to moderate guidelines (eg, dietary guidelines), divert attention and resources away from effective research, and undermine independent evidence.^49^, ^50^, ^51^ These results lead to fundamental conflicts of interest, compromising public health goals.^26^, ^52^

## Taking a Systems Approach

There is therefore an urgent need for a plurality of public health approaches to NCD prevention, integrating mixed methods from a variety of sources and disciplines^53^ to understand how our environments—or systems—might react to change or become more health promoting. Calls for a complex systems approach—one taking account of systems phenomena, such as interconnections between stakeholders, interventions, and their settings; and self‐organizing and emergent behavior, nonlinearity, and feedback loops—are becoming increasingly common in public health^53^, ^54^, ^55^, ^56^, ^57^ and, more specifically, in addressing the impact of UCIs.^58^ Such an approach has the potential to encourage the integration of mixed methods from a variety of sources and disciplines, including quantitative and qualitative traditions;^53^ to promote a better understanding of the wider political, institutional, and cultural context in which health outcomes, risk factors, and behaviors are embedded; and to help identify potential leverage points for pro–public health interventions in the system. A systems approach would facilitate shifting away from the predominantly isolated intervention thinking and to regaining traction in the governance of public health change by understanding where power currently resides.^59^ Pursuing a systems approach can thus allow action at the most crucial points in the system as well as systemic interventions that improve public health.

A complex system may be characterized by its heterogeneity (with various actors and structures at different levels); its dynamic, interactive, and adaptive nature (its ability to respond to or resist external changes, or changes in the interacting parts); and its emergent properties (arising through interactions between processes or factors that alone do not exhibit such properties).^55^, ^56^, ^60^ A “high‐functioning” or embedded system is said to have a combination of 3 important characteristics: (1) resilience, (2) self‐organization or adaptivity, and (3) hierarchy.^60^ *Resilience* is a measure of a system's ability to survive and persist in a variable environment, its ability to bounce or spring back into shape after being pressed or stretched. *Adaptivity*, or self‐organization (a system's capacity to make its own structure simpler or more complex, to diversify and evolve in response to external pressures), and *hierarchy* (arrangements of subsystems to facilitate the system's functioning) further contribute to a system's resilience.^60^ A system that is dynamic, interactive, and adaptive may be better able to respond to or resist external changes or changes in the interacting parts. It is useful to consider these properties when thinking of a public health intervention whose objective is usually to change someone's behavior. If the system, or its subsystems (eg, the food system), that a public health intervention is trying to change is so resilient that it can *absorb* that intervention and “spring back into shape,” it may in this way render the intervention ineffective.

While NCDs are often conceptualized in terms of individual‐level risk factors, we argue that they should be reframed as the product of a complex system, having been shaped by a set of interrelated systems like the processed‐food and alcohol systems. Thus the involvement of UCIs in public health policymaking can itself be thought of as a complex intervention often made up of many interacting components operating at different levels and with different possible outcomes and mechanisms and implemented in different contexts.

This complex intervention presents challenges and opportunities for using innovative methods and approaches to describe, analyze, and evaluate the various ways in which UCIs are involved in public health policies and also the (health and nonhealth) impact of that involvement. Employing a systems thinking approach could help conceptualize a public health issue as embedded in the wider political, economic, institutional, and cultural systems; reveal the underlying characteristics and relationships of systems; and show how they interrelate to produce outcomes. Systems thinking stipulates that understanding relationships among components of a system necessitates acknowledging the system's context and culture. It also enables moving away from “factors thinking” (listing factors that may influence a result) and “intervention thinking” (which recommends solutions based on isolated, discrete interventions) toward “operational thinking” (understanding how interventions work in combination and interact with their context).

Despite these advantages, the translation of systems thinking into practical applications in public health is challenging, and existing applications remain on the whole at the theoretical level.^54^, ^56^ This article theorizes that the effect of UCIs’ products and strategies on NCDs is greater than the sum of its parts. In other words, corporate influence is not just a question of linear relationships between a marketing strategy and individual consumption, or between corporate presence at a scientific conference and public health research agendas. Instead, it is a question of how these strategies come together to create a system that is resilient to public health interventions through its capacity to adapt and diversify.

## Applying a Systems Thinking Framework to NCDs’ Commercial Determinants

Next we explore the systems thinking framework proposed by Donella Meadows^61^ and examine how it can be used to analyze the commercial determinants of NCDs, and, specifically, how UCIs influence public health policy. Meadows suggests that a system can be conceptualized as a set of elements interconnected in such a way that they produce their own pattern of behavior over time and with a specific function or purpose.^60^ She proposes 12 leverage points in a system, organized into (1) elements, (2) interconnections, and (3) purpose.

*Elements* include such tangible components as individual citizens, individuals’ skills, and a system's physical structures.^56^, ^60^ The elements of a system are the most visible or concrete and thus are the easiest and most obvious point of intervention. This is therefore where most public health interventions occur;^56^ yet focusing on the system's elements is often the least effective place in which to bring about change.^61^

Meadows explains the importance of *interconnections* by referring to the following saying: “You think that because you understand ‘one’ that you must therefore understand ‘two’ because one and one make two. But you forget that you must also understand ‘and.’ ” Interconnections include physical flows (shifting objects from point A to point B) and information flows (rules, instructions), characterized by balancing or reinforcing feedback loops, and self‐organization. These are, for example, the governance, economics, and sustainability of alcohol processing. Understanding these interconnections, that is, the structures, interdependencies, and feedback within a system, can help the public health community better identify damping effects and blockages in the system, as well as barriers to effective action, and clarify where actions and changes in structure need to occur, thus leading to significant and enduring improvements.^62^

Finally, a system's *purpose* or *function* can be characterized by its goals and paradigms and include both intended responses (eg, a soft drinks company aims to increase soft drink consumption; a government health department aims to improve health outcomes) and unintended consequences. A system's operation at the level of the paradigm refers to the system's most deeply held beliefs that drive its goals, rules, and structures. Although it may be difficult for the public health community to intervene at this level, it can be very effective.^56^ This is, for example, the level at which advocates were trying to act when working on the successful introduction of minimum unit pricing for alcohol in Scotland, consistently framing alcohol policy as a multisectoral public health issue requiring a whole‐population approach.^63^

How a system and its components are defined is in many ways determined by one's perspective and interests. The predominant perspective will depend on how the *elements* are portrayed; that is, who are presented as legitimately the predominant actors, as well as their relative power within the system; whether evidence concerning the health impact of products is contested; and how *interconnections* are formed (who decides on and influences policy). Whether a system's core *function* or *purpose*, for instance, is regarded as production and sales (of a commodity) or a population's well‐being and health depends on whether a production and trade perspective (economic gain) or a health‐driven perspective (health gain) is dominant. In turn, the definition of the system and its components and how debates around it are framed determine the interventions and solutions that are considered.

### A Worked Example: Applying the Systems Thinking Framework to Analyzing Commercial Determinants of NCDs

**Figure 1:**
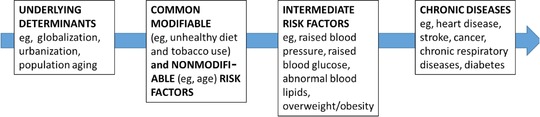
Causes of Chronic Diseases Adapted from WHO.^65^ [Color figure can be viewed at http://wileyonlinelibrary.com]

A systems approach can begin with the development of a conceptual model to encode “our beliefs about the networks of causes and effects that describe how a system operates, along with the boundary of the model (which variables are included and which are excluded) and the time horizon we consider relevant.”^64^ Nonetheless, most conceptual models, like that in Figure 1, are visually linear, illustrating the effects of certain factors along a pathway rather than showing and characterizing the interactions between factors. Such causal pathways are certainly useful in clarifying the sectors and factors of interest and also as communication tools. The main shortcoming of such illustrations is that they do not necessarily allow for thinking through how these sectors and factors might interact to produce an outcome.

**Figure 2:**
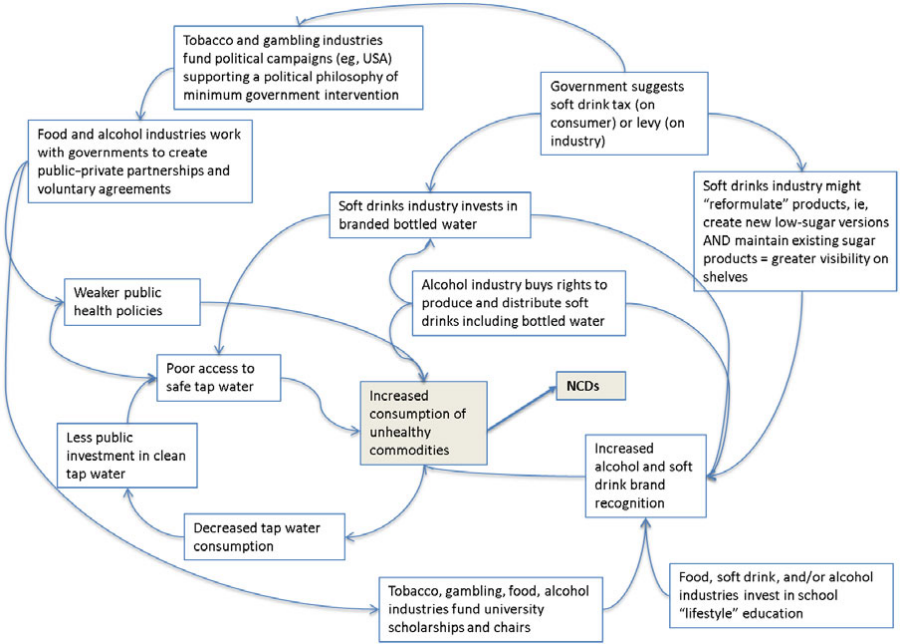
A Worked Example of a Systems Map of an “NCD‐Genic” System* *Authors’ own, based on evidence.^44^, ^50^, ^70^, ^71^, ^72^, ^73^, ^74^ [Color figure can be viewed at http://wileyonlinelibrary.com]

We next offer an example of a more complex, nonlinear, preliminary conceptual model of an “NCD‐genic” system, specifically focused on NCDs’ commercial determinants (Figure 2). Models like this can be built through approaches such as qualitative systems dynamic modeling, an innovative approach to understanding the nonlinear behavior of complex systems^66^ that has been successfully employed, for example, to analyze housing as a complex system by mapping the links between environmental, economic, social, and health outcomes^66^ or to analyze urban water management.^67^ Visualizing the system with the help of a causal loop diagram (CLD)^68^ can help unpack the individual system components and interconnections between them. CLDs can help create a concise narrative about a particular problem; they can be thought of as “sentences” constructed through the identification of key variables in a system (the “nouns”) and the explanation of the causal relationships between them via links (the “verbs”).^69^

This model is a deliberate attempt to begin to display NCD‐related system complexity, but it is by no means comprehensive. For example, a more complete version would contain advertising products to the public, the direct lobbying of politicians, and alternative solutions apart from public‐private partnerships, such as education. All the relationships or interactions the model depicts are based on evidence.

This conceptual model—while arguably more complex and less easily accessible than Figure 1—allows the depiction of both market and nonmarket strategies, as well as the highlighting of sectors or factors (like the unintended consequence of unsafe tap water) that otherwise would not be considered, even though they play an important role in NCDs. Developing such a model can thus help visualize systems’ underlying characteristics and relationships and show how they interact to produce an outcome. Through this lens, NCDs can be conceptualized as an emergent property of a complex system, rather than the results of individual factors, like lifestyle choices.

Visualizing the complex system can also help unpack its individual components (ie, the system's elements, interconnections, and functions), understand how they might contribute to or help mitigate NCDs, and identify the key research questions and methods that can help analyze them.

### Elements of an NCD‐Genic System

Although the *elements* of a system are the most visible or tangible, and thus the easiest point of intervention, they often are the least effective place to intervene.^61^ We will use the marketing of Coca‐Cola as an example. It is rarely the product itself on which consumers are made to focus, but rather the *experience* the product is meant to bring the consumer: “Ads never showed what was in Coca‐Cola, but instead what Coca‐Cola was in: pleasant, poignant scenes of everyday life.”^75^ Typically, public health research and programs try to redress the balance by emphasizing the product itself to demonstrate that it is bad for health if consumed in excess. These approaches, however, fail to acknowledge the emotional connection that the advertisers have made between the drink and imagined positive life experiences. As a result, consumers are rarely interested in the physical properties of the product itself, including any of its negative properties. Although knowing and describing the elements of a system will not explain the system's behavior,^60^ it is nonetheless essential to identify (qualify and quantify) the elements at play in order to be able to think through what interconnections might exist and why. In Figure 2, elements might include the range of actors, the latest evidence relating to market access and trade, consumption patterns, scientific research on the impact of consumption, as well as the physical arrangements to facilitate trade and consumption. Policies and programs may also be considered as elements of the system. Methods to use when identifying a system's elements and gauging their importance to the system include stakeholder mapping to identify key actors; historical analyses to understand context and culture; policy analysis to understand the scope and nature of policy commitments; analysis of industry documents and narratives via media analyses; RCTs of relevant interventions; national surveys of consumption and behaviors; systematic reviews and meta‐analyses; and logic models.

### How UCIs Interact With Others to Shape an NCD‐Genic System

How can we best analyze how a system's elements interact and therefore how to intervene effectively? Key points include understanding feedback loops (reinforcing and balancing), information flows, rules, self‐organization (the power to add, change, or evolve system structure), and power relations. A good example of the last is UCIs’ building constituencies, that is, forming alliances with others beyond their core business to create an appearance of larger support for their position.^20^, ^30^, ^76^ Sports sponsorship is a common example, with gambling companies sponsoring half of the Premier League team shirts.^77^ Other examples include Diageo's financial involvement in the National Organisation for Foetal Alcohol Syndrome.^78^

Using the feedback loop as an example of a mechanism by which change in a variable is either amplified (positive or reinforcing feedback) or lessened (negative or balancing feedback),^79^ feedback mechanisms can be used to reinforce feedback between behavioral and environmental characteristics. For example, the promotion of healthier diets might in turn create a wider demand for healthy foods.^55^ A reinforcing feedback loop enhances whatever direction of change is imposed on a system, and it can also have detrimental effects.^79^ Drawing on the example presented in Figure 2, poor access to safe tap water can contribute to increased soft drink consumption, which, in turn, might lead to decreased tap water consumption and less public investment in clean tap water. An unintended consequence might therefore be the increased marketing of bottled water by soft drink companies in the area. This also has the unintended consequence of encouraging a view in the population that the main way to protect one's family's health is to buy safe commercial products in the marketplace rather than relying on collective solutions such as supplies of safe tap water. Thus, investigating the interconnections between the system's elements can provide invaluable insights into nonlinear and unanticipated effects and can help inform the most appropriate action.^55^, ^56^ Table 1 outlines some of the key questions to ask when trying to understand a system's interconnections and what methods to use to do so.

**Table 1:** Understanding Interconnections in a System

Questions to ask when aiming to understand interconnections in a system	Potential methods
What are the causal maps/pathways/processes, and how can they help or hinder an intervention?	Logic models^80^ Tracer methods^81^, ^82^ Group model building^83^ Causal loop diagrams (as an analytic tool)^68^
What structures are in place? What processes occur? What interests are at play?	Social network analysis^84^, ^85^ Stakeholder analysis^86^, ^87^ Economic game theory Analysis of industry documents, tactics, and strategies^88^, ^89^ Analysis of policy documents Media analyses^90^
What are the feedback loops? How does the system constrain/suppress, and/or potentiate the effects of an intervention on the outcomes of interest?	Simulation‐based approaches such as Agent‐based models Longitudinal analysis Monitoring trends over time
How “resilient” is the system to change? Does the system “absorb” interventions? Is the system resistant to change?	Policy evaluations^37^, ^91^ Analysis of industry documents, tactics, and strategies

### How UCIs Operate at the Level of the Paradigm to Influence a System's Purpose

A system's purpose is the least tangible of its components, as it “is not necessarily spoken, written, or expressed explicitly.”^60^ Although often characterized by goals and paradigms, a system's purpose or function is best deduced from its behavior rather than from its stated goals (eg, a government health department may have a stated aim to improve population health outcomes but allocates little money or commitment to that goal).^60^ An effective analysis of a system's purpose or function thus includes an observation of its goals, its intended and unintended responses, and its outcomes. From a public health perspective, systems should fundamentally be designed to improve population health,^92^ with governments responsible for driving health‐protecting policies and preventive approaches. Nevertheless, and understandably, these views are seldom shared by all actors involved in shaping the system. The assumption that health is a central concern for all involved is faulty, because governments negotiate pressures and demands from a range of sectors to deliver a range of goals.

A powerful example of how a UCI operated at the level of the paradigm to influence the system's deepest held beliefs (globally, one could argue) is again the symbolism portrayed by Coca‐Cola, embodying Western open‐market values, freedom, happiness, youthful culture, and democracy.^93^ There are countless examples of how Coca‐Cola has managed to achieve this. One reported by the company itself is its decision to distribute the soft drink to East Germans a few hours after the Berlin Wall came down in 1989, signaling the beginning of a new era^94^ and market opportunities. It is reported that in the first week after the fall of the Berlin Wall, 2 million people “drank a toast to freedom with a Coke.”^94^ The soft drink is portrayed as transcending social and cultural boundaries in a familiar yet aspirational way. Andy Warhol, whose art often critiqued consumerist values, explained the supposed democratizing power of Coca‐Cola:

You can be watching TV and see Coca‐Cola, and you know that the President drinks Coke, Liz Taylor drinks Coke, and just think, you can drink Coke, too. A Coke is a Coke and no amount of money can get you a better Coke than the one the bum on the corner is drinking. All the Cokes are the same and all the Cokes are good. Liz Taylor knows it, the President knows it, the bum knows it, and you know it.^95^

When Coca‐Cola entered the Soviet Union around 1990, Pepsi had already been there for decades as a result of an early trade agreement with that country. It is said that Pepsi's early presence backfired, however, because it represented the fallen Soviet system; by contrast, Coca‐Cola was able to portray itself as representing freshness and liberation and rapidly overtook Pepsi throughout most of Eastern Europe.^96^ When thinking of intervention success, this example illustrates the importance of focusing on understanding the context (shifting political situation, what it implies culturally, and how “deepest held beliefs” may actually be changing rapidly) instead of focusing on the product itself (the taste of Pepsi or, in the case of a public health intervention, a nutrition education message on the need to curb the consumption of sugary drinks).^97^

When assessing a system's function or purpose, one can explore the behaviors (vs statements) of key actors; how an industry or brand aims to shape or tap into beliefs, knowledge, attitudes, behaviors; and why and in what target group(s) beliefs, knowledge, attitudes, and behaviors are shaped.

## Discussion

### The Value of Taking a Systems Approach

Drawing on Meadows's systems thinking framework, we examined how a systems perspective can be applied to analyze commercial determinants of NCDs and specifically how UCIs influence public health policy. We demonstrated how taking a systems approach to understanding commercial determinants of NCDs helps identify more clearly how UCIs market their products, gain agency over policy and politics, and legitimize their increasing presence in public health decision making. A systems approach also shows that the effect of a given intervention depends on other conditions in the system as well as the interconnections between the elements of that system.^55^ This approach also stipulates that understanding these interconnections requires acknowledging the system's context and culture.^55^, ^56^, ^97^

Applying a systems approach to addressing NCDs clearly shows that such a complex health issue is embedded in the wider economic, political, institutional, and cultural system rather than merely being the result of individual lifestyle choices. Current public health research still concentrates mainly on a system's elements rather than the interconnections within it, and this is beginning to reveal its intrinsic limitations. Changing individual variables rarely changes a system's behavior.^60^ Ultimately the central goal of public health actions will be to achieve sustainability of public health gains in the context of a dynamic system, and a systems approach can support this goal: sustainability includes the intervention's adaptability, the persistence of changes made, the ongoing adoption of new changes, scalability (diffusion across settings), and reach (diffusion across population groups, crossing cultural and other boundaries).^98^, ^99^

We demonstrated that a systems analysis may start with producing a worked example of a causal loop diagram of the NCD‐genic system, which includes knowledge and assumptions about the fundamental processes that may be involved. The development of a conceptual model of the system can be a prelude to developing new evidence‐based interventions and evaluating them, evaluating other changes in the system, like the introduction of new policies that might—intentionally or not—produce changes in rates of NCDs, or synthesizing and understanding existing evidence to direct further interventions and future research. It is important to note that there are several systems approaches and related methods, many of which are starting to be applied to public health policy analysis.^54^

A systems thinking approach can therefore help clarify the complexities of NCDs by drawing out their interpersonal, community, and intersectoral dynamics for generating solutions; generating new hypotheses and identifying gaps in empirical data; analyzing intended and unintended consequences; and identifying the networks and actors influencing decision making in health as well as the policy levers that can be used to address public health problems.^57^ For example, this approach can be a powerful mechanism to understand effective countervailing forces against UCIs’ influence in public health policy, such as the crucial role of civil society organizations in the imposition of an effective soft drink tax in Mexico.^100^

Explicit and early recognition of the role of UCIs in future causal models of NCDs and in the development of policies and regulatory frameworks (such as the Framework Convention on Tobacco Control)^101^ will be essential. As Green noted, faulty thinking leads to failed planning and action.^102^ Moreover, developing ways to manage UCIs’ influence on policies through long‐term mechanisms, which transcend political cycles, will be an essential strategy.^103^

## Conclusion

This article contributes to the growing literature on applying systems thinking to public health problems. Applying a systems lens to a complex example such as the commercial determinants of NCDs illustrates its potential to help avoid linear thinking and to identify interconnections and complexities as a starting point for further research and action. The questions and methods we proposed are meant to be useful for researchers by identifying the types of knowledge that help characterize a system and how its components work together to generate NCDs (or other complex public health problems). Principles of systems thinking may be applicable to NCDs as well as to other complex problems, and they should now be tested and refined for other public health challenges.
